# Maternal High-Fat and High-Salt Diets Have Differential Programming Effects on Metabolism in Adult Male Rat Offspring

**DOI:** 10.3389/fnut.2018.00001

**Published:** 2018-03-07

**Authors:** Stephanie A. Segovia, Mark H. Vickers, Claudia J. Harrison, Rachna Patel, Clint Gray, Clare M. Reynolds

**Affiliations:** ^1^Liggins Institute, University of Auckland, Auckland, New Zealand

**Keywords:** developmental programming, high-fat diet, high-salt diet, dietary sodium, metabolic inflammation, insulin sensitivity

## Abstract

Maternal high-fat or high-salt diets can independently program adverse cardiometabolic outcomes in offspring. However, there is a paucity of evidence examining their effects in combination on metabolic function in adult offspring. Female Sprague Dawley rats were randomly assigned to either: control (CD; 10% kcal from fat, 1% NaCl), high-salt (SD; 10% kcal from fat, 4% NaCl), high-fat (HF; 45% kcal from fat, 1% NaCl) or high-fat and salt (HFSD; 45% kcal from fat, 4% NaCl) diets 21 days prior to mating and throughout pregnancy and lactation. Male offspring were weaned onto a standard chow diet and were culled on postnatal day 130 for plasma and tissue collection. Adipocyte histology and adipose tissue, liver, and gut gene expression were examined in adult male offspring. HF offspring had significantly greater body weight, impaired insulin sensitivity and hyperleptinemia compared to CD offspring, but these increases were blunted in HFSD offspring. HF offspring had moderate adipocyte hypertrophy and increased expression of the pre-adipocyte marker *Dlk1*. There was a significant effect of maternal salt with increased hepatic expression of *Dgat1* and *Igfb2*. Gut expression of inflammatory (*Il1r1, Tnfα, Il6*, and *Il6r*) and renin–angiotensin system (*Agtr1a, Agtr1b*) markers was significantly reduced in HFSD offspring compared to HF offspring. Therefore, salt mitigates some adverse offspring outcomes associated with a maternal HF diet, which may be mediated by altered adipose tissue morphology and gut inflammatory and renin–angiotensin regulation.

## Introduction

The incidence of obesity and related non-communicable diseases, including type 2 diabetes, cardiovascular diseases, and some cancers continues to rise. Although these conditions have a genetic component, the rapid rate at which these conditions have increased underscores the influence of a rapidly changing lifestyle. In parallel with the increase in obesity and related non-communicable diseases is a shift toward consumption of a Western-style diet, often composed of processed foods which are high in saturated fat and sodium (1–4). Despite the relevance to current diets, there is a paucity of mechanistic studies examining the effects of high fat (HF) and high salt in combination, and results remain inconclusive. Some studies in mice suggest a synergistic adverse effect of fat and salt on cardiac, endothelial, and vascular markers (5, 6). Furthermore, high salt in conjunction with HF may exacerbate hepatic inflammation and oxidative stress and the subsequent progression of non-alcoholic steatohepatitis (7). In contrast, Weidemann et al. have shown in mice that dietary sodium prevented weight gain in HF feeding, despite no difference in food intake or metabolic rate (8). This was suggested to be due to reduced digestive efficiency by suppression of the renin–angiotensin system (RAS). Consequently, the reported effects of diets high in both fat and salt are conflicting.

The Developmental Origins of Health and Disease hypothesis proposes that an adverse early life environment, such as suboptimal maternal nutrition, can program obesity and chronic disease in offspring (9). Both maternal HF and maternal high-salt diets are independently associated with adverse programming effects in offspring. Maternal HF (obesogenic) diets program obesity and metabolic syndrome in offspring (10, 11). Maternal high-salt diets program increased blood pressure in offspring and adverse alterations in vascularity, contributing to increased cardiovascular disease risk (12, 13). However, the combined impact of a maternal HF and salt diet on developmental programming of offspring health has not been thoroughly assessed. There is evidence to suggest that maternal low-grade inflammation may mediate programming effects in offspring (14–16). HF diets are independently implicated in promoting low-grade inflammation (17), and the addition of high salt to a HF diet has been reported to exacerbate inflammatory processes (7). Therefore, it might be expected that a maternal HF and high-salt diet in combination would intensify adverse programming effects in offspring.

We have previously demonstrated altered placental function and offspring early life growth in response to maternal HF and/or high-salt diets (18, 19). Intrauterine growth restriction, followed by accelerated postnatal growth, is associated with later life obesity and metabolic syndrome (20). At day 18 of pregnancy, male fetuses from HF and high-fat and salt (HFSD) fed dams were significantly smaller than fetuses from control (CD) fed dams (18). There were no significant differences in male birth weights (unpublished findings). However by postnatal day 21 male HF offspring were significantly heavier than male HFSD offspring. These findings suggest that a maternal HFSD diet during pregnancy and lactation may blunt an adverse early life growth trajectory in offspring compared to offspring from HF dams. Male HF and HFSD placentas at day 18 of pregnancy had increased expression of *Tnf*α, and there was a significant increase in expression of glucose and amino acid transporters in the HF-exposed groups (19). We speculated that the increased nutrient transport may be a compensatory response due to HF diet-induced placental insufficiency. However, whether these alterations have long-term implications on metabolic function in adult offspring is unclear. Therefore, we aimed to examine how a maternal HF and high-salt diet fed independently and in combination would impact inflammation and markers involved in regulation of the gut–adipose–liver axis in adult male offspring.

## Materials and Methods

### Animal Model

**Figure 1:**
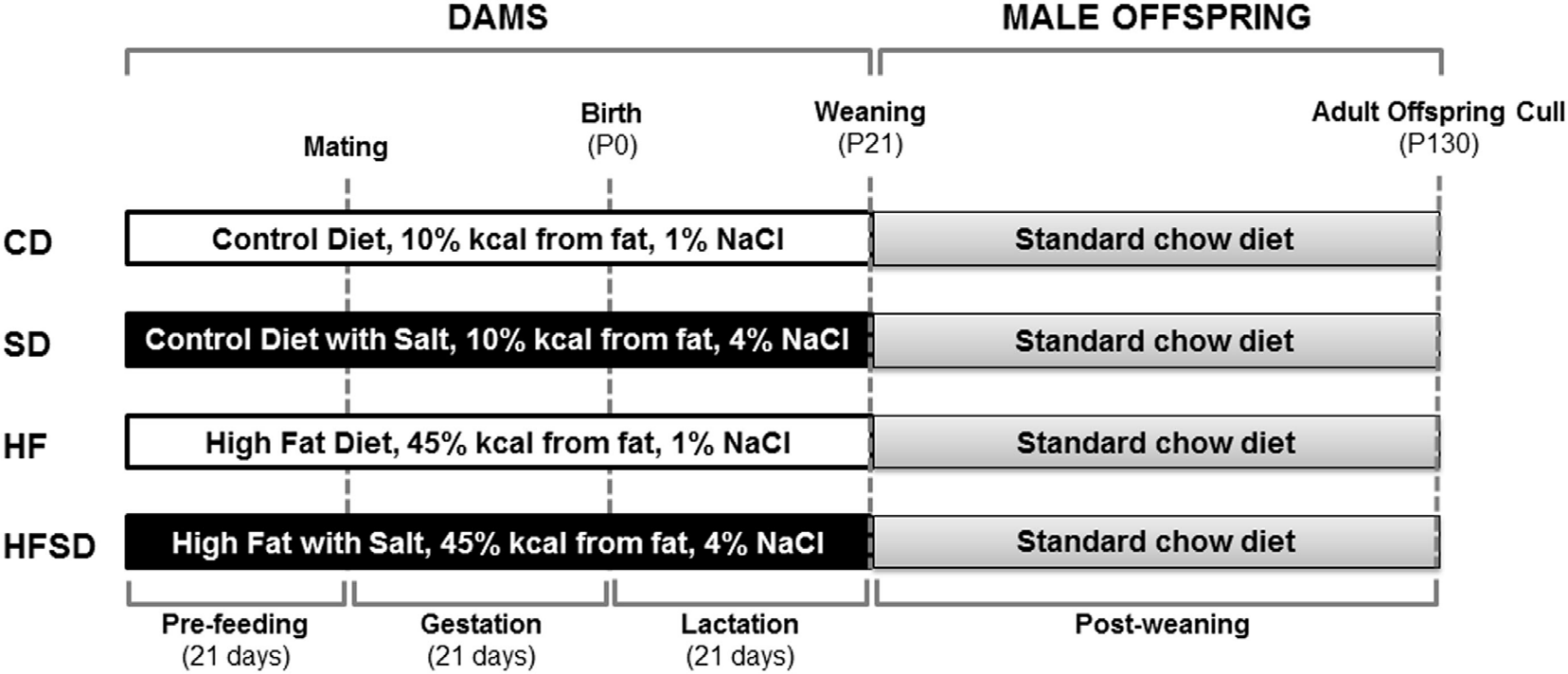
Overview of experimental design. Female Sprague-Dawley rats consumed either a CD, SD, HF, or HFSD diet *ad libitum* for 21 days prior to mating and throughout pregnancy and lactation. Male offspring were weaned onto a standard chow diet *ad libitum*. At postnatal day 130 (P130), male offspring were culled for plasma and tissue collection for analysis.

The procedures described were approved by the Animal Ethics Committee at the University of Auckland and were performed in accordance with relevant guidelines and regulations. The exper-imental protocol was performed as previously described by Gray et al. (21) (Figure 1). Animals were housed at 22°C with a 12 h light: 12 h dark cycle. 28 female virgin Sprague Dawley rats were obtained at weaning and fed a standard chow diet *ad libitum* until day 90 (Harlan Teklad Global Diets; Diet 2018). Rats were then randomly assigned to one of four experimental diets and fed *ad libitum* for 21 days prior to mating and throughout gestation and lactation. Experimental groups (*n* = 7/group) were fed either a: purified control (CD; 10% kcal from fat, 1% NaCl), purified high salt (SD; 10% kcal from fat, 4% NaCl), purified high fat (HF; 45% kcal from fat, 1% NaCl), or purified high fat and high salt (HFSD; 45% kcal from fat, 4% NaCl) diet and are further detailed by Reynolds et al. (19) (Research Diets, New Brunswick, NJ, USA). Female rats (115 ± 2 days) were time-mated using an estrous cycle monitor (EC-40, Fine Science Tools, San Francisco, CA, USA), and pregnancy was confirmed by detection of spermatozoa following vaginal lavage. In order to maintain standardized nutrition until weaning, litter size was randomly adjusted to eight pups (four males and four females), and unused pups were killed by decapitation. Data presented in this paper are for male offspring, and female offspring were used in an independent unrelated study. Post-weaning, offspring consumed a standard chow diet *ad libitum* up to day 130. At day 130, animals were fasted overnight and killed by decapitation under pentobarbitone anesthesia (intraperitoneal injection; 60 mg/kg). Tissues were dissected, weighed and snap frozen and stored at −80°C or fixed in formalin. Trunk blood was collected in heparinized vacutainers (Becton Dickinson, Franklin Lakes, NJ, USA) and plasma stored at −20°C until analysis.

### Plasma Analysis

Plasma was analyzed for insulin and leptin by commercial rat-specific ELISAs (Crystal Chem, Chicago, IL, USA). The cytokines interleukin (IL)-1β, IL-6, and tumor necrosis factor α (TNFα) were analyzed by Quantikine ELISA (R&D Systems; Minneapolis, MN, USA). Plasma was also analyzed for glucose, free fatty acids, triglycerides, low-density lipoprotein cholesterol (LDL), high-density lipoprotein cholesterol (HDL), total cholesterol, and lactate dehydrogenase by Hitachi 902 autoanalyzer (Hitachi High Technologies Corporation, Tokyo, Japan).

### Isolation of Stromal Vascular Fraction (SVF)

Gonadal adipose tissue was immediately dissected from culled animals and placed in sterile phosphate-buffered saline. 1 g of adipose tissue was finely minced and placed in digestion buffer (Krebs ringer bicarbonate buffer; 0.155 M NaCl, 0.2 M NaHCO_3_, 0.1 M KH_2_PO_4_, 0.12 M KCl, 0.05 M CaCl_2_, 0.1 M MgS0_4_, and 1 M HEPES, pH adjusted 7.35–7.45; 4% BSA, 2 mg/mL collagenase). The mixture was incubated in a shaking water bath at 37°C for 45 min and then filtered. Flow through was spun, and the SVF pellet was collected and stored in TRI Reagent (Sigma-Aldrich, St. Louis, MO, USA) for subsequent analysis.

### Histological Analysis

Adipocyte histology was performed as previously described (22). Retroperitoneal adipose tissue samples (*n* = 7/group) were fixed in 10% neutral buffered formalin, paraffin embedded, and then sectioned (8 µm) using a Leica RM 2135 rotary microtome (Leica Instruments, Nussloch, Germany). Standard hematoxylin and eosin staining was performed. Slides were viewed under light microscopy at 10 × objective, and digital images were acquired with NIS Elements-D software (Nikon 800, Tokyo, Japan). Images were blindly and manually analyzed by ImageJ 1.46v software (US National Institutes of Health, Bethesda, USA). Four representative fields of view were analyzed from each section to determine average adipocyte area.

### Gene Expression Analysis

RNA was isolated from retroperitoneal adipose tissue and the SVF with TRI Reagent (Sigma-Aldrich, St. Louis, MO, USA). RNA was isolated from the liver and the upper gut (duodenum) using the RNeasy Mini Kit (Qiagen, Hilden, Germany). RNA concentration was determined using the NanoDrop 1000 Spectrophotometer (Thermo Fisher Scientific Inc., Wilmington, DE, USA). Reverse transcription was performed using the High-Capacity cDNA Reverse Transcription Kit (Applied Biosystems, Warrington, UK). Quantitative real-time polymerase chain reaction (RT-qPCR) analysis was performed on the ABI 7900HT Fast RT-qPCR System using Sequence Detection System 2.4 software to quantify mRNA expression using TaqMan Fast Advanced Master Mix and pre-designed TaqMan Gene Expression Assays (Applied Biosystems, Warrington, UK; Table S1 in Supplementary Material). To control for variability between samples, the relative amounts of the genes were normalized to peptidylprolyl isomerase A (*Ppia*), hypoxanthine–guanine phosphoribosyltransferase 1 (*Hprt1*), and/or glyceraldehyde 3-phosphate dehydrogenase (*Gapdh*) expression. The comparative CT method (2^–ΔΔCT^) was used to analyze data (23).

### Statistical Analysis

Data were graphed using Prism 6 software (GraphPad Software Inc., La Jolla, CA, USA), and statistical analysis was performed using SigmaPlot 12.5 (Systat Software Inc., San Jose, CA, USA). Data were analyzed by two-way analysis of variance, with maternal HF and maternal salt as factors. When data failed normality (Shapiro–Wilk test), it was log transformed. Holm–Sidak *post hoc* tests were performed where indicated for multiple comparisons testing between groups. Differences between groups were considered significant at *P* < 0.05. All data are presented as mean ± SEM.

## Results

### A Maternal HF Diet Programmed an Adverse Metabolic Phenotype in Adult Male Offspring, Which Was Not Exacerbated by the Addition of Salt to the Maternal Diet

There were no significant differences in male offspring birth weights (data not shown). The physiological and metabolic profiles of adult male offspring are presented in Table 1. There were significant effects of a maternal HF diet and maternal salt intake on adult male offspring weights. Offspring from HF fed mothers were significantly heavier compared to all other off-spring. Although the same pattern was mirrored in the retroperitoneal adipose tissue percentage, it did not reach statistical significance. However, there was an increase in retroperitoneal adipose tissue weight in the HF-exposed groups. There were no significant differences in fasting glucose, but there were significant interactions in insulin, and the homeostasis model assessment of insulin resistance (HOMA-IR), a proxy for insulin resistance. HF offspring had significantly increased insulin and HOMA-IR compared to CD offspring, indicating reduced insulin sensitivity which was normalized in the HFSD offspring. The same pattern was observed with leptin concentrations. There were no significant differences in the plasma concentrations of the cytokines IL-1β and TNFα. However, there was a significant interaction in IL-6, with SD, HF, and HFSD groups having reduced concentrations compared to CD. There was a significant effect of maternal HF on increasing plasma triglycerides, but no significant differences in LDL, HDL, or total cholesterol.

**Table 1:** Adult male offspring metabolic profile.

	CD	SD	HF	HFSD	Maternal HF	Maternal salt	Interaction
Weight (g)	624.36 ± 19.86	600.24 ± 16.32^+^	725.27 ± 14.59*	635.79 ± 30.81^+^	*P* = 0.004	*P* = 0.014	NS
Retroperitoneal adipose tissue (g)	18.72 ± 1.39	13.63 ± 1.61^+^	24.31 ± 3.19	19.42 ± 3.24	*P* = 0.033	*P* = 0.058	NS
Retroperitoneal adipose tissue (% body weight)	3.00 ± 0.20	2.28 ± 0.25	3.33 ± 0.41	2.95 ± 0.41	NS	NS	NS
Liver (% body weight)	2.69 ± 0.05	2.62 ± 0.09	2.60 ± 0.13	2.57 ± 0.09	NS	NS	NS
Leptin (ng/mL)	3.73 ± 1.07	9.68 ± 2.33	25.54 ± 7.41*	5.15 ± 2.02^+^	NS	NS	*P* = 0.002
Fasting glucose (mmol/L)	9.36 ± 0.40	9.03 ± 0.32	9.43 ± 0.41	9.03 ± 0.23	NS	NS	NS
Fasting insulin (ng/mL)	1.42 ± 0.24	3.43 ± 0.84	4.40 ± 1.37*	1.38 ± 0.23^+^	NS	NS	*P* = 0.013
HOMA-IR	0.57 ± 0.08	1.48 ± 0.40	1.91 ± 0.62*	0.56 ± 0.10^+^	NS	NS	*P* = 0.015
IL-1β (ρg/mL)	31.72 ± 2.87	31.49 ± 2.65	26.93 ± 2.65	27.12 ± 2.87	NS	NS	NS
IL-6 (ρg/mL)	196.92 ± 22.12	80.89 ± 22.12*	99.33 ± 20.48*	83.15 ± 22.12*	*P* = 0.04	*P* = 0.006	*P* = 0.032
TNFα (ρg/mL)	8.08 ± 4.23	12.61 ± 3.57	19.45 ± 3.57	11.81 ± 3.86	NS	NS	NS
Free fatty acids (mmol/L)	2.32 ± 0.35	2.19 ± 0.32	1.39 ± 0.30	2.49 ± 0.35	NS	NS	NS
Triglycerides (mmol/L)	0.81 ± 0.11	0.68 ± 0.10	1.04 ± 0.09	0.89 ± 0.11	*P* = 0.042	NS	NS
LDL (mmol/L)	0.27 ± 0.04	0.27 ± 0.04	0.24 ± 0.04	0.26 ± 0.04	NS	NS	NS
HDL (mmol/L)	1.92 ± 0.19	1.84 ± 0.17	1.65 ± 0.16	1.72 ± 0.19	NS	NS	NS
Total cholesterol (mmol/L)	1.84 ± 0.16	1.92 ± 0.15	1.87 ± 0.14	1.85 ± 0.16	NS	NS	NS
Lactate dehydrogenase (U/L)	403.50 ± 94.05	577.17 ± 94.05	503.50 ± 81.45	696.50 ± 94.05	NS	*P* = 0.056	NS

Plasma was analyzed by ELISA [leptin, insulin, interleukin (IL)-1β, IL-6, and tumor necrosis factor (TNF) α] or Hitachi autoanalyzer [glucose, free fatty acids, triglycerides, low-density lipoprotein cholesterol (LDL), high-density lipoprotein cholesterol (HDL), total cholesterol, and lactate dehydrogenase]. HOMA-IR (homeostasis model assessment of insulin resistance) was calculated using the following calculation: [fasting glucose (mmol/L) × fasting insulin/22.5]. Data were analyzed by two-way analysis of variance with Holm–Sidak post hoc analysis, where *P < 0.05 compared to CD and ^+^P < 0.05 compared to HF. NS indicates the effect is not statistically significant.

### Maternal Diets High in Salt Impact Adipocyte Size and Adipogenesis in Adult Male Offspring

**Figure 2:**
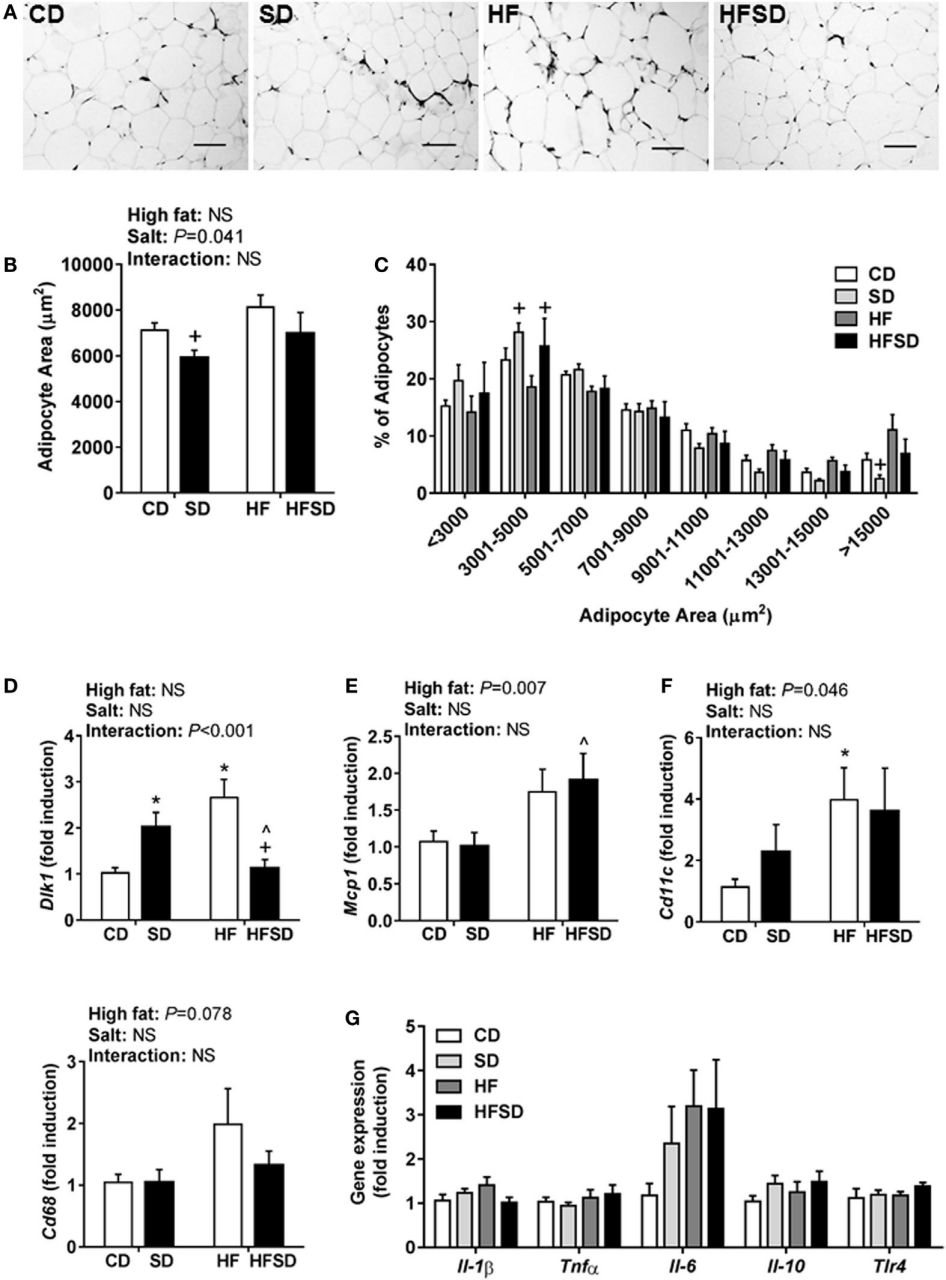
Adipocyte morphology and adipogenic and inflammatory markers in retroperitoneal adipose tissue of adult male offspring. Fixed retroperitoneal adipose tissue was sectioned and stained with hematoxylin and eosin (*n* = 7/group). Four representative images were blindly analyzed per animal in Image J 1.48v. **(A)** Representative images from each group; scale bar represents 100 µm. **(B)** Average adipocyte area and **(C)** adipocyte area according to distribution in size. Retroperitoneal adipose tissue gene expression of **(D)** *Dlk1*; **(E)** *Mcp1*; **(F)** *Cd11c*; and **(G)** *Il-1*β; *Tnfα*; *Il-6*; *Il-10;* and *Tlr4* (*n* = 7/group). Data were analyzed by two-way analysis of variance, with *post hoc* Holm–Sidak tests for multiple comparisons. Data are expressed as mean ± SEM, where *P < 0.05 vs CD, ^*P* < 0.05 vs SD, and ^+^*P* < 0.05 vs HF.

**Figure 3:**
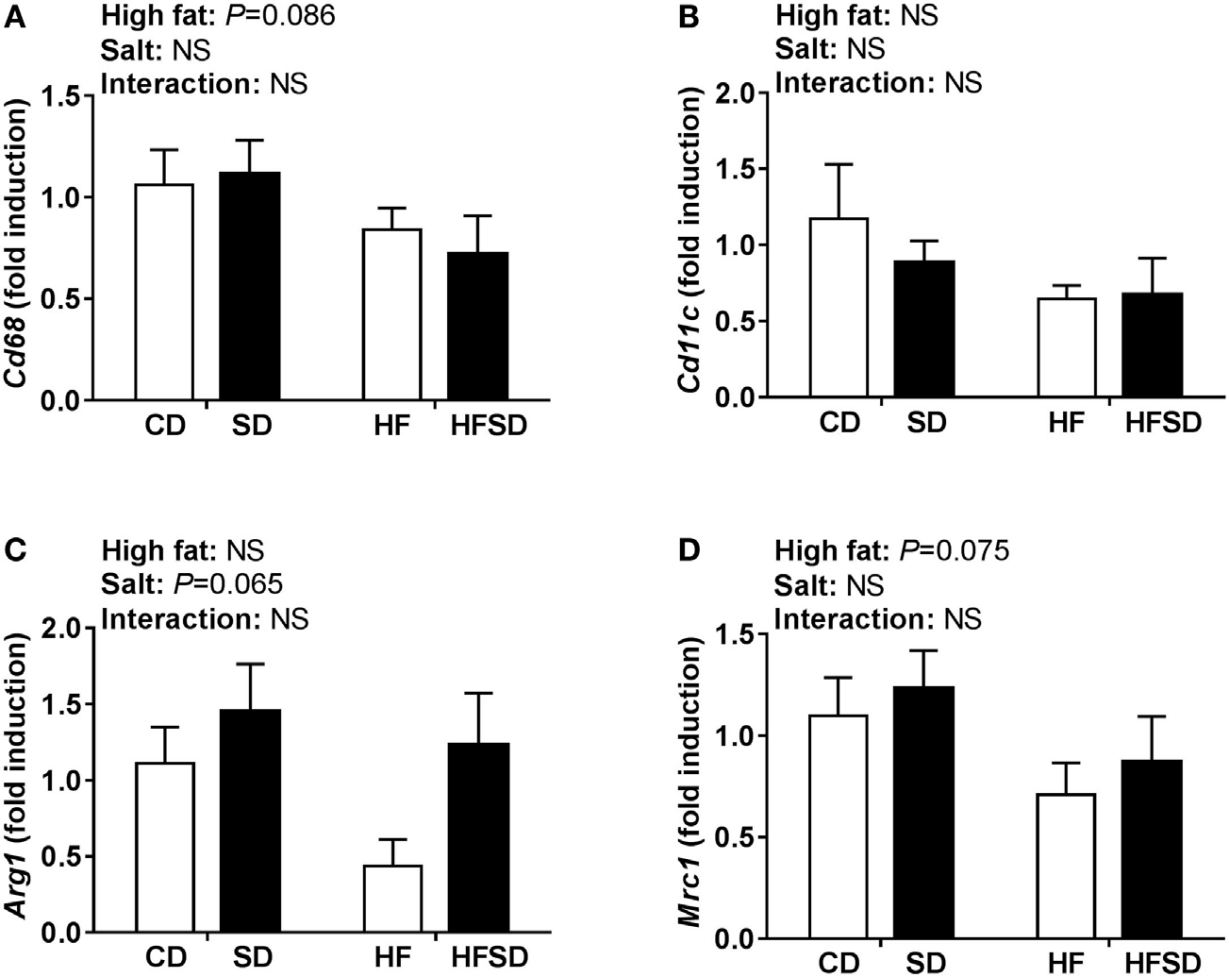
Expression of macrophages markers in the stromal vascular fraction from gonadal adipose tissue. **(A)** *Cd68*; **(B)** *Cd11c*; **(C)** *Arg1;* and **(D)** *Mrc1* gene expression determined by RT-qPCR (*n* = 7/group). Data were analyzed by two-way analysis of variance. Data are expressed as mean ± SEM.

Consistent with the changes in body weight and the trend in retroperitoneal adipose tissue percentage, we observed a significant effect of maternal salt on average adipocyte size in offspring, with significantly reduced average retroperitoneal adipocyte size in the SD group compared to the HF group (Figures 2A,B). When adipocytes were graphed according to the percentage of total adipocytes falling within a certain range (Figure 2C), the SD and HFSD groups had significantly more adipocytes with an area between 3,001 and 5,000 µm^2^ than the HF group. This was due to the HF group having a greater number of adipocytes with an area >15,000 μm^2^ compared to the SD group. These data suggest moderate adipocyte hypertrophy in HF offspring, with a greater proportion of smaller adipocytes in the SD group. Given the changes in adipocyte morphology, we examined gene expression in the retroperitoneal adipose tissue. There was a significant interaction in *Dlk1*, a pre-adipocyte marker and inhibitor of adipogenesis (Figure 2D). *Post hoc* analysis revealed a significant increase in SD and HF groups compared to CD, and a significant reduction in the HFSD group compared to SD and HF groups. There were significant effects of a maternal HF diet on the expression of the macrophage-related genes *Mcp1* (Figure 2E) and *Cd11c* (Figure 2F). There were no significant differences in retroperitoneal adipose tissue expression of the inflammatory markers *Il-1*β, *Tnf*α, *Il-6, Il-10*, or *Tlr4* (Figure 2G). There were no statistically significant differences in macrophage-related markers (*Cd68, Cd11c, Arg1*, and *Mrc1*) in the SVF isolated from gonadal adipose tissue (Figure 3).

### Maternal Salt Impacts Hepatic Gene Expression

**Figure 4:**
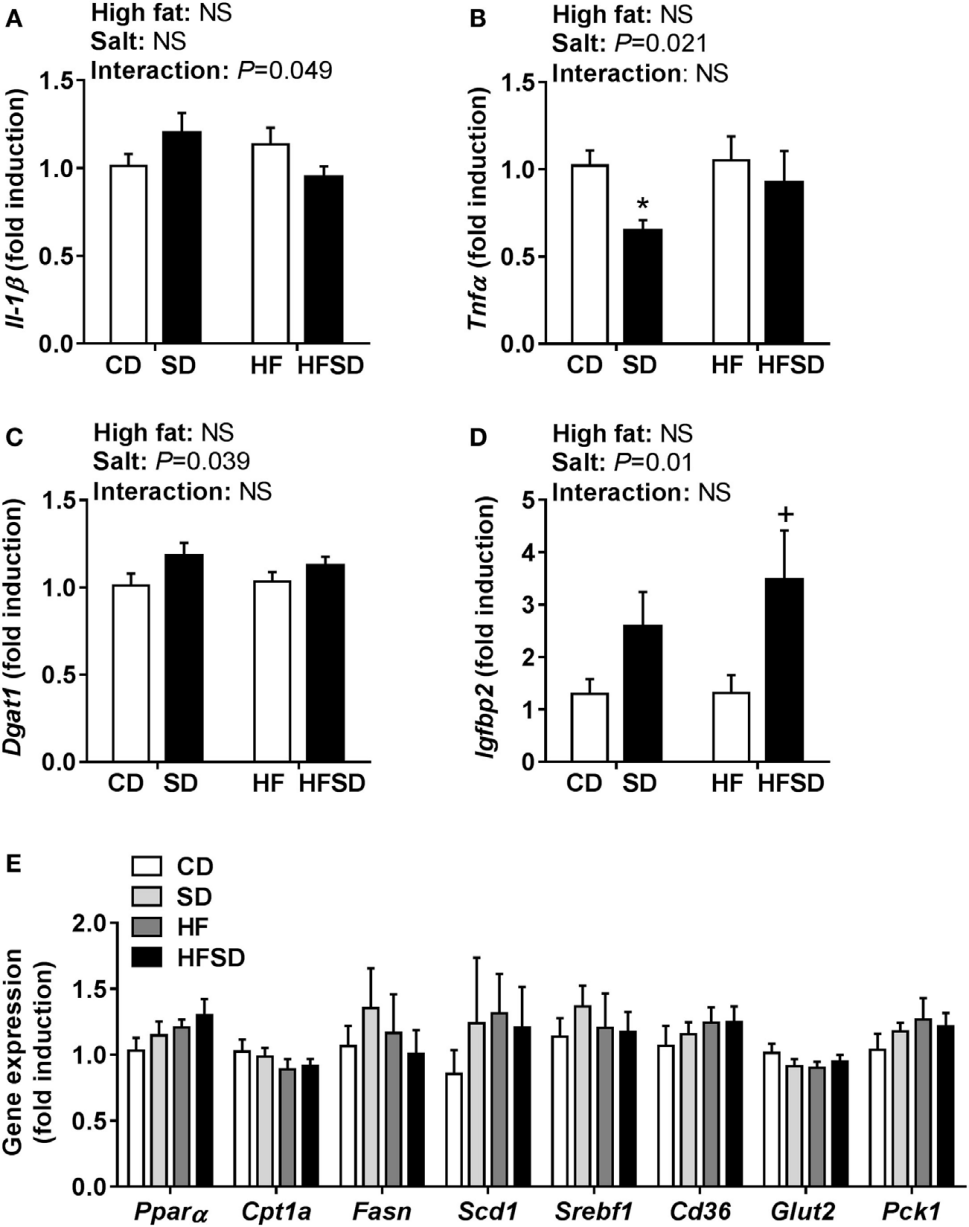
Hepatic gene expression of inflammatory cytokines and markers associated with insulin resistance. **(A)** *Il-1*β; **(B)** *Tnf*α; **(C)** *Dgat1*; **(D)** *Igfbp2*; and **(E)** *Ppar*α; *Cpt1a*; *Fasn*; *Scd1*; *Srebf1*; *Cd36*; *Glut2;* and *Pck1* gene expression determined by RT-qPCR (*n* = 7/group). Data were analyzed by two-way analysis of variance, with *post hoc* Holm–Sidak tests for multiple comparisons. Data are expressed as mean ± SEM, where *P < 0.05 vs CD and ^+^*P* < 0.05 vs HF.

To gain insight into the differences in insulin sensitivity in off-spring, we examined hepatic gene expression. There was a significant interaction in *Il-1*β expression (Figure 4A). There was a significant effect of maternal salt intake on *Tnf*α expression, with *post hoc* analysis showing a reduction in SD compared to CD (Figure 4B). *Dgat1*, the gene encoding the enzyme responsible for triglyceride synthesis, was overall significantly increased in the maternal salt groups (Figure 4C). There were no significant differences in the expression of *Igfbp1*, a gene encoding a protein that binds to insulin-like growth factors (data not shown), but *Igfbp2* was significantly increased by maternal salt intake, with a significant increase in the HFSD group compared to the HF group (Figure 4D). There were no significant differences in hepatic expression of markers related to lipid (*Ppar*α, *Cpt1a, Fasn, Scd1, Srebf1*, and *Cd36*) or glucose (*Glut2* and *Pck1*) transport and metabolism (Figure 4E).

### Maternal HF and HFSD Diets Differentially Impacted Inflammatory, Lipid Transporter, and RAS Receptor Markers in Offspring Gut

**Figure 5:**
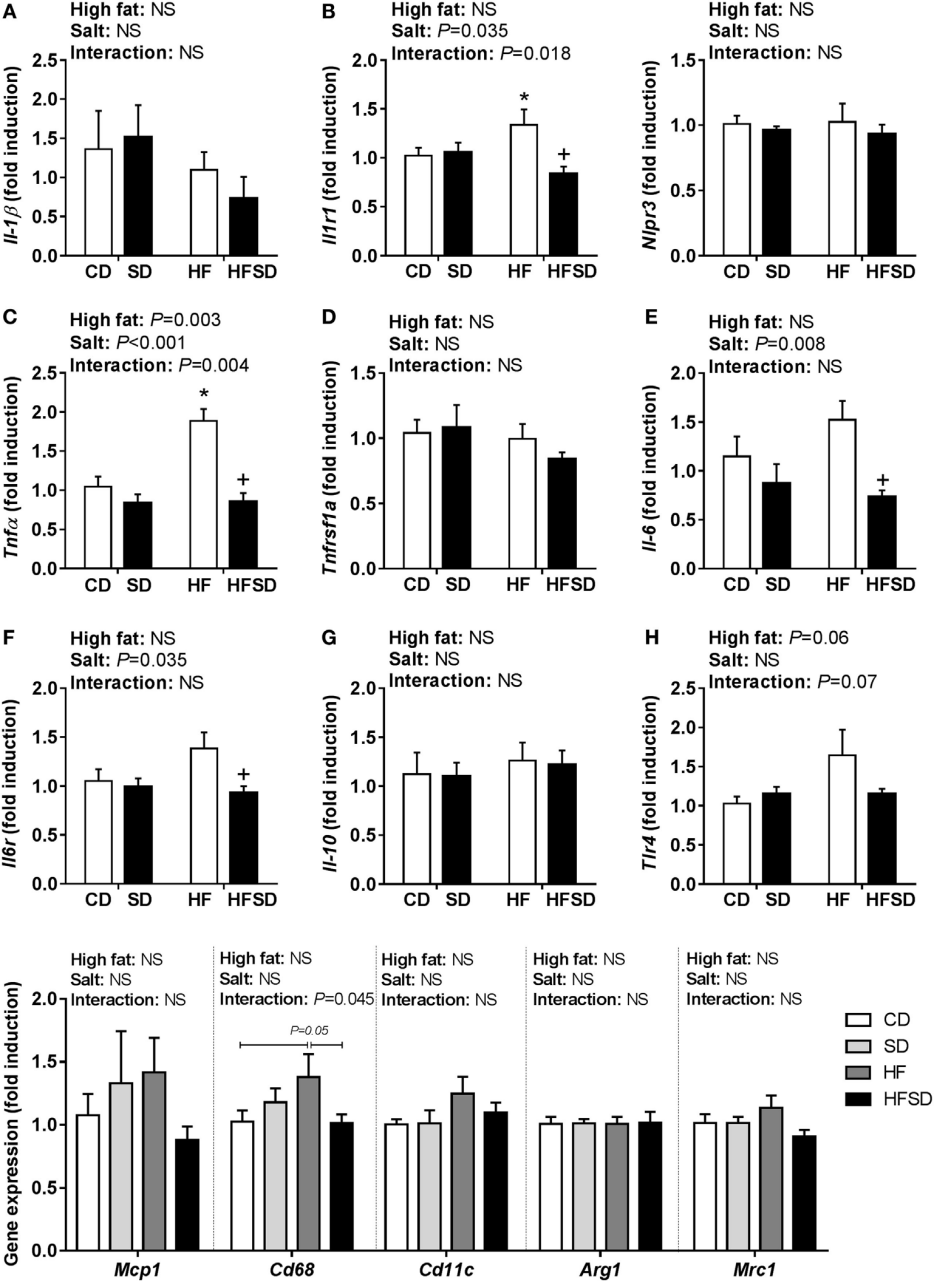
Expression of pro-inflammatory markers in male offspring gut. **(A)** *Il-1*β; **(B)** *Il1r1*; **(C)** *Tnf*α; **(D)** *Tnfrsf1a*; **(E)** *Il-6*; **(F)** *Il6r*; **(G)** *Il-10;* and **(H)** *Tlr4* gene expression determined by RT-qPCR (*n* = 7/group). Data were analyzed by two-way analysis of variance, with *post hoc* Holm–Sidak tests for multiple comparisons. Data are expressed as mean ± SEM, where *P < 0.05 vs CD and ^+^*P* < 0.05 vs HF.

**Figure 6:**
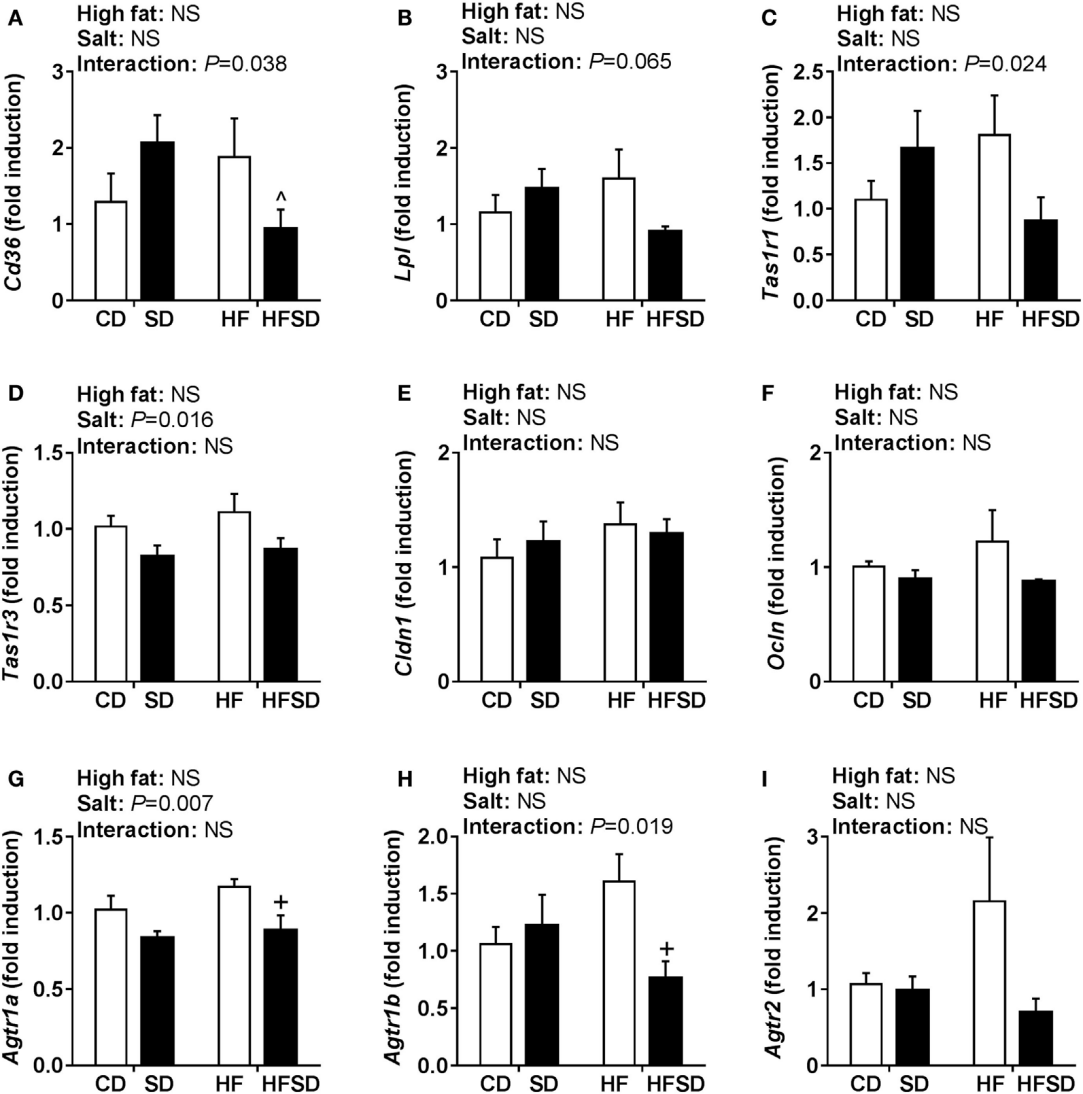
Gut gene expression of lipid transporters, taste receptors, and RAS receptors. **(A)** *Lpl*; **(B)** *Cd36*; **(C)** *Tas1r1*; **(D)** *Tas1r3*; **(E)** *Cldn1*; **(F)** *Ocln*; **(G)** *Agtr1a*; **(H)** *Agtr1b*; and **(I)** *Agtr2* gene expression determined by RT-qPCR (*n* = 7/group). Data were analyzed by two-way analysis of variance, with *post hoc* Holm–Sidak tests for multiple comparisons. Data are expressed as mean ± SEM, where ^*P* < 0.05 vs HFSD and ^+^*P* < 0.05 vs HF.

Given the emerging role of altered low-grade inflammation in the gut in models of developmental programming, we assessed inflammatory markers in the gut. There were no significant differences in *Il-1*β expression (Figure 5A), although there was a significant interaction in the expression of its receptor *Il1r1*, with *post hoc* analysis showing a significant increase in HF compared to CD and reduction in HFSD compared to HF (Figure 5B). This was mirrored in the expression of *Tnf*α (Figure 5C), however, not in its receptor *Tnfrsf1a* (Figure 5D). There were significant effects of maternal salt on *Il-6* and its receptor *Il-6r*, with significantly reduced expression in HFSD groups compared to HF groups (Figures 5E,F). There was no difference in IL-10 and TLR4 expression between groups (Figures 5G,H). We also examined markers of lipid transport, taste receptors, and barrier function in the gut. There was a trend in the expression of *Lpl* (Figure 6A), although this did not reach statistical significance (*P* = 0.065). There was a significant interaction in *Cd36* expression, with significantly reduced expression in the HFSD group compared to SD (Figure 6B). There was a significant interaction in expression of *Tas1r1* and significant effect of maternal salt on *Tas1r3* (Figures 6C,D). There were no significant differences in the expression of the tight junction proteins *Cldn1* and *Ocln* (Figures 6E,F). The angiotensin II receptors *Agtr1a, Agtr1b*, and *Agtr2* were assessed. There was a significant effect of maternal salt on *Agtr1a* and significant interaction in *Agtr1b*, with *post hoc* analysis showing reduced expression of these receptors in the HFSD groups compared to HF (Figures 6G,H). There were no significant alterations in *Agtr2* (Figure 6I).

## Discussion

Despite the relevance to current Western diets, the effect of maternal exposure to a diet high in both saturated fat and salt on programming of offspring health is largely unknown. Therefore, we assessed the impact of maternal diets high in either fat or salt, and in combination, on male adult offspring metabolism in a small animal model. We found that a maternal HFSD diet resulted in significantly reduced weight, increased insulin sensitivity, and reduced circulating leptin concentrations in offspring compared to those exposed to a maternal HF diet alone. However, in the absence of more comprehensive physiological assessments, it is unclear where the alterations in body weight arose from. Given leptin concentrations, we speculate that the differences in body weight were due to differences in total body fat percentage. Although this was not necessarily evident in the retroperitoneal adipose tissue, increased fat storage in other fat depots such as subcutaneous is a possibility. Collectively, these findings suggest that a maternal HFSD diet prevents some of the adverse programming effects associated with a maternal HF diet in adult male offspring.

Given the physiological differences we observed between groups in the current study, we assessed retroperitoneal adipose tissue morphology and gene expression. The adipose tissue can secrete a wide range of hormones and cytokines which can contribute to IR, including leptin, IL-1β, TNFα, IL-6, and MCP1 (24). Systemic and adipose tissue low-grade inflammations are key features of obesity-related IR (25). In addition, M1 macrophages that express *Cd11c* on their surface infiltrate the adipose tissue and further promote inflammation (26–28). We observed that a maternal HF diet increased the expression of macrophage markers *Mcp1* and *Cd11c* similarly in both HF and HFSD groups (29, 30). These findings are in line with DeClercq et al. who recently reported that feeding mice a HF and high-salt diet (60% kcal from fat, 4% NaCl) did not exacerbate the increase in adipose tissue cytokines or macrophages seen in mice fed a HF diet alone (30). We did not observe significant differences in circulating IL-1β and TNFα or the expression of *Il-1*β, *Tnf*α, *Il-6*, or *Il-10* in the retroperitoneal adipose tissue. The reduction in circulating IL-6 in SD, HF, and HFSD groups compared to CD may be attributable to its pleiotropic role in metabolism (31). Although the lack of systemic and adipose tissue inflammation may appear to conflict with the phenotype, others have reported that IR can occur in the absence of increased cytokines. Independent of inflammation, enlarged adipocytes may be capable of inducing IR (31). In a mouse model of HFD-induced obesity and IR, the mesenteric, epididymal, perirenal, and subcutaneous depots all displayed adipocyte hypertrophy, but there was depot-specific macrophage infiltration (in mesenteric and epididymal depots) and inflammation (in the mesenteric depot only) (32).

In obesity, the increase in adipose tissue mass occurs through adipocyte hypertrophy and/or hyperplasia (33). Hypertrophy occurs as the adipose tissue attempts to meet the increased capacity for lipid storage resulting in an increase in the volume of existing adipocytes. It is generally associated with negative outcomes including hypoxia, infiltration of macrophages, and induction of inflammation in the adipose tissue (34). Further-more, adipocyte hypertrophy is independently associated with IR and hyperleptinemia (35). We observed marked differences in the expression of *Dlk1* in the retroperitoneal adipose tissue. *Dlk1* is a pre-adipocyte marker, which is downregulated when adipocytes undergo differentiation (36). Compared to metabolically healthy obese individuals, metabolically unhealthy obese individuals had increased expression of *DLK1* in omental adipose tissue, concomitant with increased adipocyte size and increased macrophage number (37). The authors speculated that the increased expression of *DLK1* in unhealthy adipose tissue prevented pre-adipocyte differentiation, making mature adipocytes hypertrophic and perhaps leading to hypoxia and macrophage recruitment. In line with this, offspring in the HF group had increased expression of *Dlk1* and histological analysis also suggests moderate adipocyte hypertrophy in the HF group. Since *Dlk1* inhibits pre-adipocyte proliferation and differentiation (38), the increased *Dlk1* expression in HF offspring may indicate a disturbance in the generation of new mature adipocytes to cope with this demand. In contrast, the HFSD group had significantly lower expression of *Dlk1* compared to both SD and HF groups, on par with expression in the CD group. This normalization of *Dlk1* expression may explain how insulin sensitivity and leptin concentrations were similar in CD and HFSD groups.

There is a clear link between obesity and IR in the liver, thus we assessed gene expression in the liver. *Dgat1* encodes the gene for the enzyme that catalyzes the last step of triglyceride production, and *Dgat1* null mice are protected from the development of diet-induced obesity and IR (39). The slight increase in *Dgat1* expression in SD and HFSD groups was not reflected in the concentrations of circulating triglycerides. *Igfbp1* and *Igfbp2* are binding proteins for insulin-like growth factors and regulate its biological activity in a number of ways. In particular, low circulating concentrations of IGFBP2 are associated with both obesity and T2DM. Wheatcroft et al. demonstrated that mice overexpressing IGFBP2 are protected against these conditions through inhibition of adipogenesis, preventing hepatic steatosis and reducing circulating leptin concentrations (40). IGFBP2 is regulated by leptin and can improve insulin sensitivity in animals who are leptin resistant (41). Therefore, SD and HFSD offspring may have been partially protected from reduced insulin sensitivity through altered regulation of leptin and its downstream target, *Igfbp2*. In offspring exposed to maternal undernutrition, we reported increased circulating leptin concentrations, in the absence of altered hepatic *Igfbp2* expression, which may indicate hepatic leptin resistance (41). Following this, in the present study HF offspring had increased circulating leptin with no change in hepatic *Igfbp2* expression, which could also implicate hepatic leptin resistance in HF but not SD and HFSD groups. However, more recent work has demonstrated that leptin’s physiological effects occur independent of IGFBP2 (42). Therefore, in the absence of more substantial differences in the liver, the physiological relevance of the modest alterations we observed in gene expression is unclear. This may indicate that adipose–liver crosstalk is not heavily involved in the differences in insulin sensitivity observed in this experimental paradigm at this time point. However, as animals were culled at P130, which may be considered relatively young in the rat (43, 44), we cannot exclude the possibility that these subtle alterations may be an early indication of impairment that may worsen with age. Indeed aging reduces insulin secretion in offspring exposed to an adverse maternal nutritional environment and other rodent studies have examined rat offspring exposed to maternal obesity at up to postnatal day 450 (45).

Emerging evidence supports a major role of the gut in mediating the low-grade inflammation and IR induced by obesity. The leaky gut hypothesis proposes that increased intestinal permeability promotes increased release of bacterial LPS, which can bind to TLR4 and promote inflammatory cytokine secretion (46, 47). Li et al. showed that diet-induced obese mice had increased IL-1β and IL-12p40 concentrations in both the colon and the surrounding mesenteric adipose tissue, but not in the perigonadal or subcutaneous adipose tissue (48). Furthermore, in a mouse model of dextran sulfate sodium-induced colitis, hepatic, subcutaneous, and mesenteric adipose tissue inflammation was also induced. Kawano et al. have recently reported that short-term (4 weeks) HF feeding in C57BL/6J mice promotes inflammation and macrophage infiltration in the colon, which precede inflammatory changes in the adipose tissue (49). Furthermore, HFD challenged mice with *Mcp1* deletion (specific to either macrophages or intestinal epithelial cells) demonstrated improved insulin sensitivity concomitant with reduced adipose tissue inflammation (50). However, macrophage gene expression in the small intestine in response to a HFD was not altered. These findings illustrate that intestinal inflammation can potentially be both a consequence and cause of inflammation associated with obesity. We found significantly increased gut expression of the inflammatory markers *Il1r1* and *TNF*α in the HF group compared to CD, which was blunted in the HFSD group. There was a significant reduction in *Il6* and *Il6r* in the HFSD group compared to HF. Following the same pattern as the cytokines, there was a trending interaction in gut expression of *Tlr4*. These findings support the impaired insulin sensitivity found in the HF group only and highlight importance of the maternal diet in programming inflammatory regulation in the offspring gut. Our findings also suggest that gut inflammation may contribute to impaired insulin sensitivity and may precede adipose tissue inflammation. However, further research is required to elucidate if altered macrophage infiltration in the small intestine occurs in a similar manner to the colon. There are several hypotheses proposed to explain how inflammation is propagated to or from the gut, including altered intestinal permeability. Although we did not detect any changes in the expression of the tight junction proteins *Ocln* and *Cldn1*, there are a number of tight junction proteins and at least 23 other claudins (51) expressed throughout the intestine which were not examined, and therefore, we cannot rule out the involvement of altered gut permeability.

Weidemann et al. have recently shown that the addition of salt to a HF diet prevented weight gain by reducing digestive efficiency through sodium-induced reduction in markers of the RAS (8). These regulatory systems were assessed to determine whether they were involved in the unexpected programming effects observed in response to a maternal HFSD diet. CD36 is a protein responsible for the uptake and absorption of fatty acids, and intestinal expression of *Cd36* is increased in mice by a HF diet (52). Interestingly, CD36 in peripheral blood mononuclear cells is elevated in individuals with T2DM and is associated with circulating inflammatory markers. While we observed elevated *Cd36* expression in SD and HF groups, expression in the HFSD group was normalized to a level similar to that of the CD group. This may indicate reduced absorption of fatty acids in the HFSD compared to SD and HF groups and may contribute to the reduced weight and improved insulin sensitivity seen in the HFSD group.

Recently, the regulation of processes in the gut has emerged for its potential role in health. In particular, it has been shown that gut taste receptors are responsible for nutrient sensing and can affect secretion of gut hormones involved in digestion, including GLP1 and PYY (53, 54). TAS1R1 and TAS1R3 form a heterodimer which detects umami taste, and TAS1R3 can also form a heterodimer with TAS1R2 to detect sweet taste (55, 56). We were recently the first to report that offspring at weaning from HF-fed mothers have increased expression of *Tas1r1* and reduced expression of *Tas1r3* in the gut compared with offspring from CD mothers (57). In line with these findings, there was a significant interaction between maternal HF and salt on gut *Tas1r1* expression in the gut of adult male offspring. In contrast, there was a significant effect of maternal salt on reducing *Tas1r3* expression. However, as the precise mechanisms underpinning the regulation of taste receptors in the gut remains poorly understood and we did not measure circulating concentrations of GLP1 and PYY, the impact of these findings is unclear.

The RAS is typically known for its roles in cardiovascular homeostasis and fluid regulation. However, in recent years, components of the RAS have been shown to be expressed in multiple peripheral tissues, and it is clear that the RAS contributes to a range of physiological effects outside its classically described roles. Increased activation of the RAS is associated with obesity and T2DM, while blocking the RAS can be protective against diet-induced obesity and development of T2DM (58). Although components of the RAS have been shown to be expressed in the intestine (59), the impact of altered regulation of the RAS in the intestine still remains poorly understood. Weidemann et al. have shown that dietary sodium suppresses digestive efficiency and prevents HF diet-induced weight gain (8). These effects were attributed to the AT_2_ receptor as both antagonism and gene knock-out resulted in suppressed digestive efficiency. However, their model involved a global reduction of the AT_2_ receptor. It is not clear if local reductions in RAS components in the intestine alone (as we observed in the HFSD group compared to the HF group) can impact digestive efficiency and weight gain.

In conclusion, we have demonstrated that the addition of high salt to a maternal HF diet did not exacerbate the adverse effects of a maternal HF diet on regulation of metabolism in adult offspring. Rather, a maternal HFSD diet prevented some of the programmed effects in offspring as a consequence of a maternal HF diet, including impaired insulin sensitivity and hyperleptinemia. In line with this finding, a maternal HFSD diet prevented a maternal HF diet-induced increase in the key lipid transporter *Cd36* and inflammatory markers in the gut. However, what is unclear from the present study is how this occurs. We have previously demonstrated in this model that dams consuming the HFSD diet during pregnancy gained significantly less weight than dams on the HF diet, although there were no significant differences in cumulative caloric consumption between the two groups (18). Indeed, others have recently shown that the addition of high salt on top of a HF diet prevented weight gain compared to a HF diet alone. Adult offspring from dams on a high-salt diet during pregnancy and lactation have been reported to have reduced body weight, which was not attributable to differences in birth weight (21). Since differences in body weight emerged during lactation, the authors speculated that changes in the composition or amount of milk consumed by offspring could contribute to these effects (21). In line with this, we observed no differences in birth weights of male offspring (unpublished findings). However, male HF offspring had accelerated growth in the pre-weaning period compared to all other male offspring. Therefore, although speculative, it is possible that there is an interaction between high salt and HF, when consumed in combination, which directly alters maternal metabolism to prevent weight gain (e.g., basal metabolic rate and nutrient absorption) and/or impact lactation and therefore promotes adverse programming effects on offspring. However, it is clear that the specific dietary interactions between salt and saturated fat and how they affect body weight and metabolism are poorly defined and warrant further investigation.

## Ethics Statement

This study and procedures described were carried out in accordance with the recommendations and regulations of the Animal Ethics Committee at the University of Auckland.

## Author Contributions

Conceived and designed experiments: SAS, CMR, MHV and CG. Performed experiments: SAS, CMR, RP, CG and CJH. Wrote manuscript: SAS. Edited manuscript: CMR and MHV.

## Conflict of Interest Statement

The authors declare that the research was conducted in the absence of any commercial or financial relationships that could be construed as a potential conflict of interest.

## Abbreviations

ANOVA, analysis of variance; CD, purified control diet group; HDL, high-density lipoprotein cholesterol; HF, purified high-fat diet group; HFSD, purified high-fat and high-salt diet group; HOMA-IR, homeostasis model assessment of insulin resistance; IL, interleukin; LDL, low-density lipoprotein cholesterol; RAS, renin–angiotensin system; RT-qPCR, reverse transcription quantitative real-time polymerase chain reaction; SD, purified high-salt diet group; SVF, stromal vascular fraction; TNFα, tumor necrosis factor α.
