# Supplementary material for: Maternal High-Fat and High-Salt Diets Have Differential Programming Effects on Metabolism in Adult Male Rat Offspring
Source: Front Nutr. 2018 Mar 7;5:1. doi: 10.3389/fnut.2018.00001 (PMC5845870; doi:10.3389/fnut.2018.00001)
Supplement: Supplementary file 1 [file Data_Sheet_1.PDF]

Supplementary Material

Maternal High Fat and High Salt Diets Have Differential Programming Effects on Metabolism in Adult Male Rat Offspring

Stephanie A Segovia[1], Mark H Vickers[1], Claudia J Harrison[1], Clint Gray[1], Clare M Reynolds[1]*

*** Correspondence:** Dr Clare Reynolds: c.reynolds@auckland.ac.nz

Supplementary Table S1. TaqMan probes. Details of pre-designed TaqMan probes used in qPCR (Applied Biosystems).

Gene Symbol	Gene Name	Assay ID
Agtr1a	Angiotensin II receptor, type 1a	Rn02758772_s1
Agtr1b	Angiotensin II receptor, type 1b	Rn02132799_s1
Agtr2	Angiotensin II receptor type 2	Rn00560677_s1
Cd11c	Integrin subunit alpha X	Rn01511082_m1
Cd36	Cluster of differentiation 36 or fatty acid translocase	Rn02115479_g1
Cldn1	Claudin 1	Rn00581740_m1
Dgat1	Diacylglycerol o-acyltransferase 1	Rn00584870_m1
Dlk1	Delta-like 1 homolog	Rn00587011_m1
Gapdh	Glyceraldehyde 3-phosphate dehydrogenase	Rn01775763_g1
Hprt1	Hypoxanthine-guanine phosphoribosyltransferase 1	Rn01527840_m1
Igfbp1	Insulin-like growth factor binding protein 1	Rn00565713_m1
Igfbp2	Insulin-like growth factor binding protein 2	Rn00565473_m1
Il-1β	Interleukin 1 beta	Rn00580432_m1
Il1r1	Interleukin 1 receptor, type I	Rn00565482_m1
Il-6	Interleukin 6	Rn01410330_m1
Il6r	Interleukin 6 receptor	Rn00566707_m1
Lpl	Lipoprotein lipase	Rn00561482_m1
Mcp1	Monocyte chemoattractant protein 1	Rn00580555_m1
Ocln	Occludin	Rn00580064_m1
Ppia	Peptidylprolyl isomerase A	Rn00690933_m1
Tas1r1	Taste receptor, type 1, member 1	Rn01516038_m1
Tas1r3	Taste receptor, type 1, member 3	Rn00590759_g1
Tnfrsf1a	Tumor necrosis factor receptor superfamily, member 1a	Rn01492348_m1
Tnfα	Tumor necrosis factor alpha	Rn99999017_m1
